# Supplementary material for: Evidence for Epistatic Interaction between HLA-G and LILRB1 in the Pathogenesis of Nonsegmental Vitiligo
Source: Cells. 2023 Feb 15;12(4):630. doi: 10.3390/cells12040630 (PMC9954564; doi:10.3390/cells12040630)
Supplement: Supplementary file 1 [file cells-12-00630-s001.zip › cells-2053498-supplementary.pdf]

Supplementary data

Supplementary Table 1: Significant second-order interactions identified by MB-MDR.

SNP1	SNP1 location	SNP2	SNP2 location	betaH	WH	betaL	WL	Wmax	*p*-value
rs2114511	synonymous - *LILRB1*	rs9380142	3’UTR - *HLA-G*	0.9899	9.081	−10.347	7.476	9.081	0.013
rs61739244	synonymous - *LILRB1*	rs9380142	3’UTR - *HLA-G*	0.9811	8.864	−0.7897	5.411	8.864	0.021
rs1061684	synonymous - *LILRB1*	rs9380142	3’UTR - *HLA-G*	0.9320	7.646	NA	NA	7.646	0.026
rs148624633	missense - *LILRB1*	rs9380142	3’UTR - *HLA-G*	0.7986	5.935	−0.9103	6.974	6.974	0.027
rs28405793	synonymous - *LILRB1*	rs148624633	missense - *LILRB1*	20.721	6.816	NA	NA	6.816	0.028
rs61738512	missense - *LILRB1*	rs9380142	3’UTR - *HLA-G*	0.7432	5.157	−0.9374	7.394	7.394	0.029
rs383369	missense - *LILRB2*	rs373032	synonymous - *LILRB2*	0.9112	7.334	NA	NA	7.334	0.030
rs373032	synonymous - *LILRB2*	rs386056	missense - *LILRB2*	0.9291	7.762	NA	NA	7.762	0.031
rs2114511	synonymous - *LILRB1*	rs72492280	5’URR – *HLA-G*	10.407	6.795	NA	NA	6.795	0.031
rs366337	synonymous - *LILRB2*	rs2114511	synonymous - *LILRB1*	0.9433	5.931	NA	NA	5.931	0.031
rs373032	synonymous - *LILRB2*	rs2114511	synonymous - *LILRB1*	11.179	7.521	NA	NA	7.521	0.033
rs9380142	3’UTR - *HLA-G*	rs1632950	5’URR – *HLA-G*	10.081	7.893	−11.585	5.547	7.893	0.034
rs2114511	synonymous -*LILRB1*	rs12722482	missense – *HLA-G*	0.9749	6.380	−0.5995	3.056	6.380	0.034
rs2114511	synonymous - *LILRB1*	rs61739244	synonymous - *LILRB1*	0.8824	4.515	−0.6454	2.929	4.515	0.034
rs9380142	3’UTR - *HLA-G*	rs17179101	3’UTR - *HLA-G*	0.8581	6.834	−0.8586	5.986	6.834	0.035
rs1061684	synonymous - *LILRB1*	rs2114511	synonymous - *LILRB1*	11.535	6.019	NA	NA	6.019	0.044
rs386056	missense - *LILRB2*	rs41557518	frameshift - *HLA-G*	14.267	6.071	NA	NA	6.071	0.047
rs73938625	5’UTR - *LILRB2*	rs9380142	3’UTR - *HLA-G*	0.7843	5.729	−0.6923	4.261	5.729	0.047
rs73055442	missense -*LILRB2*	rs9380142	3’UTR - *HLA-G*	0.8324	6.433	−0.6544	3.809	6.433	0.048
rs9380142	3’UTR - *HLA-G*	rs41557518	frameshift - *HLA-G*	0.7617	5.404	−0.8583	5.985	5.985	0.048

p-value ≤ 0.05 was considered significant.
